# Sleep quality, stress level and COVID-19 in university students; the forgotten dimension

**DOI:** 10.5935/1984-0063.20210011

**Published:** 2022

**Authors:** Eptehal Dongol, Kerollos Shaker, Ahmed Abbas, Ahmed Assar, Mohamed Abdelraoof, Emad Saady, Amr Hassan, Omar Youssef, Mohamed Essam, Maysa Mahmoud, Guy Leschziner

**Affiliations:** 1 South Valley University, Qena Faculty of Medicine, Sleep disorders unit - Qena - Egypt.; 2 South Valley University, Qena Faculty of Medicine - Qena - Egypt.; 3 Qena Student Research Association (QSRA) - Qena - Egypt.; 4 Menoufia University, Faculty of Medicine - Shebin El-Kom - Menoufia - Egypt.; 5 Mansoura University, Faculty of Medicine - Mansoura - Egypt.; 6 King’s College London, sleep disorders center - London - United Kingdom.

**Keywords:** Undergraduate Students, Sleep Quality, Insomnia, Fear, Coronavirus

## Abstract

**Introduction:**

COVID-19 is a novel pandemic that has had a profound impact on global physical and psychological health. We aimed to investigate the impact of COVID-19 on stress, sleep quality, and insomnia among South Valley University students in Egypt during the quarantine period.

**Material and Methods:**

A questionnaire, including the Pittsburgh sleep quality index, the insomnia severity index, the perceived stress scale and COVID-19 fear index was distributed to the undergraduate students through the online platforms of South Valley University during the period of 1st to 15th June 2020.

**Results:**

Of a total respondent sample of 2,474 students, 24.5% had high-perceived stress levels, 31.3% had clinical insomnia, and about 80% were identified as generally poor sleepers by the PSQI. Being female, having a chronic disease, having a sleep disorder before the quarantine, or consuming caffeine were the main factors associated with high stress levels, clinical insomnia, and poor sleep quality. Also, levels of fear of COVID-19 were higher among people with high stress levels, clinical insomnia, and poor sleep quality.

**Conclusion:**

Considerable levels of stress and poor sleep quality were identified among undergraduate university students during the pandemic/home isolation period. The effect was more obvious among certain demographic groups and among the students who scored higher in the fear of COVID-19 scale.

## INTRODUCTION

On the 31^st^ of December 2019 in Wuhan city, the WHO China Country Office reported the first coronavirus disease (COVID-19) case, which began to spread rapidly from China to the rest of the world. According to the WHO, the novel COVID-19 was declared as a pandemic on the 11^th^ of March 2020. It was not until the beginning of March that it was first reported in Egypt. By the 24^th^ of January 2021, 161,143 laboratory-confirmed COVID-19 cases with 8,902 deaths have been reported in Egypt^41^.

Previous studies have indicated an increase in levels of anxiety among the public when a COVID-19-like pandemic occurs, such as severe acute respiratory syndrome (SARS); where posttraumatic stress disorder (PTSD) and depression were prevalent, in 28.9% and 31.2%, respectively, of respondents to a web-based survey to examine the psychological effects of quarantine on the individuals in Toronto, Canada^20^. Another study showed that SARS and H1N1 swine flu were related to psychological side effects due to quarantine or fear from infection and this lead to symptoms of depression that may be associated with sleep problems^25^. Similarly, since the emergence of COVID-19, there have been a lot of significant changes in all aspects of life, which have had negative impacts on mental health and sleep quality^5^. Insomnia is a very common sleep complaint^10^. It occurs due to either falling asleep with difficulty, or inability to maintain sleep for a sufficient time^23^. Recently, according to the third edition of the international classification of sleep disorders, chronic insomnia disorder diagnosis should include a report of problems in initiation or maintenance of sleep in the presence of adequate circumstances to sleep and occurrence of daytime consequences. At least three times per week for three months^34^. According to previous studies, insomnia among undergraduate students ranges between 9.5% to 27%^17,37^. Among undergraduate students, sleep deprivation and insomnia may be associated with a reduced ability of learning and memory reduction, which in turn reduces their academic performance^12,30^.

Most studies addressing the psychological effect of the COVID-19 have focused primarily on the healthcare workers or those who have been directly affected by the virus^21,42^. Generally, there is an anticipated upsurge of psychiatric illnesses that can be attributed to the COVID-19 pandemic due to the anxiety about the ambiguity of the situation with the associated health and financial problems, and also due to the isolation and the massive disruption of routines^26^.

As university students generally show higher incidences of mental disorders compared to their peers of the same age^8^, it can be expected that the current pandemic has an impact on their mental health. Most of the students’ education has been affected by the closure measures taken by the different governments in response to the pandemic^16^, the thing that led to massive learning disruptions and decreased access to proper education. And even though many institutions relied on online education, the poor infra-structures have rendered the process defective^29^, which in turn can raise students anxiety and worries about their studies and future employment^15^, in addition to their concerns on the health of their family^44^. Studies on the correlation between the COVID-19 pandemic and sleep quality among university students are limited in Egypt. Thus in this study, we aimed to assess the level of fear of COVID-19, stress level, insomnia, and sleep quality among the undergraduate students and examine the correlation between the COVID-19 related variables and the stress level, insomnia, and sleep quality.

## MATERIAL AND METHODS

### Study design

The study was a cross-sectional study that aimed to assess the levels of stress, insomnia, and the quality of sleep among college students in South Valley University, Egypt, during the period of COVID-related lockdown, and their relationship to fear of COVID and students’ demographics. Ethical approval was obtained from the ethical committee in Qena Faculty of medicine before starting dissemination of the questionnaire.

### Setting

The study was conducted via an online questionnaire that was distributed on South Valley University-related online educational platforms.

### Participants

All undergraduate students enrolled in South Valley University in all majors were eligible and invited to participate in the study by filling the online questionnaire. The population of interest is students that live in Upper Egypt (South Valley, Qena, Luxor, and Hurghada). The gender distribution is 60% females and 40% males. 15% of them study medical majors, 10% study scientific non-medical majors, and 75% study non-scientific majors.

### Variables measured in the study

The primary aim of the study was to measure the level of stress, the level of insomnia, and the sleep quality among the students. Secondary aims of the study were to examine whether the levels of stress, insomnia, and sleep quality were associated with demographics or related to the fear of COVID-19.

### Methods for measurement of the study variables

The final version of the questionnaire distributed in the online platforms consisted of 6 sections as follows:

First section: contained questions about the students’ general demographic characters (gender, residence, type of college, and year of study).

Second section: contained direct questions about the students’ health, sleep, and COVID-related demographics (having a chronic disease or a family member with a chronic disease, having a sleep-related problem before COVID, and whether the sleep habits differed after, and their direct relationship to COVID and caffeine and smoking consumption).

Third section: contained the perceived stress scale, which is a validated tool to measure the level of stress in the past period via 10 questions answered in a 5 Likert-scale including (never, rarely, sometimes, fairly often, and very often), each question had a score of 0 to 4 giving a final range of 0 to 40 with higher scores indicating higher levels of stress. We used the following cutoff points (0-13, low stress), (14-26, moderate stress), (27-40, high stress)^14^.

Fourth section: contained the insomnia severity index, which assesses the degree of insomnia via seven questions, each contains choices on a 5 Likert-scale scored (0 to 4) giving a final score range of 0 to 28 with higher scores indicating higher insomnia levels. We used the following recommended cutoff points: (0-7 = no clinically significant insomnia, 8-14 = sub-threshold insomnia, 15-21 = clinical insomnia (moderate severity), 22-28 = clinical insomnia (severe)), in the analysis the numbers were reduced to clinical insomnia (more than 15) and sub-clinical insomnia (less than 15)^9^.

Fifth section: contained the Pittsburgh sleep quality index (PSQI), which assesses the sleep quality via 9 items (questions) that are further divided into 7 components, each component takes a final score of 0 to 3, giving a final range of 0 to 21 to the final PSQI score with higher scores indicating poorer sleep quality. We used the recommended cutoff point of 5 to recognize the presence of poor sleep quality^11^.

Sixth section: containing the recently developed COVID-19 fear index scale that measures the levels of fear with seven questions each answered on a Likert-scale of 1 to 5 giving rise to a total score ranging from 7 to 35. The higher the score, the higher the fear level^1,6^.

### Sampling and sample size calculation

We used convenience-sampling method to acquire the responses from the participants via online distribution of the survey. We used the following equation n=z ^2^P(1-P)/d^2^^31^. Under a 95% CI, 50% response distribution and .05 margin of error, a sample of 384 participants can be considered as a minimal sample to represent the population of South Valley University students (n=50,000). However, due to the limitations of convenience sampling and online surveying of the potential participants, we included a design effect (DE) factor in the equation. DE is the ratio of the variance of the estimate observed with a certain type of sampling, to the expected variance of the estimate had the sample been collected using simple random sampling (SRS). For some respondent-driven sampling studies, it was recommended to be around three to four^39,40^. So, we used a design effect of 3 to 4 multiplied by the minimal sample size calculated by the previous equation as a correction factor to adjust the sample size. Finally, a minimum sample of 1,152 to 1,536 participants was considered to be the representative sample.

### Data collection and handling

The data was collected during the period of 1^st^ June to 15^th^ June 2020, which was during the period of lockdown in Egypt, which started on 15^th^ March and was ended gradually 20^th^ June. During the two weeks of data collection, Egypt underwent partial lock-down from 6 p.m. to 6 a.m. with a pause of all educational activities within the educational institutions and its replacement with online education for all the students in all majors, with a mean number of 1,200 to 1,500 new cases and 40 to 50 deaths per day. A team of data collectors was formed to distribute the questionnaire in the online study platforms of all colleges in South Valley University (social media platforms and Yammer).

### Statistical analysis

Descriptive statistics (frequencies and percentages) were used to describe the students’ demographic characters and their levels of perceived stress, their levels of insomnia, and their sleep quality. Chi-square analysis was used to compare the perceived stress levels, insomnia levels, and sleep quality with the students’ general demographics and COVID-related demographics. To test the association between the primary study outcomes (stress, insomnia, and sleep quality) and the fear of COVID-19 as a separate measure, normality of the distribution of the fear of COVID-19 scores between the different categories of stress, insomnia, and sleep quality was tested using the Shapiro-Wilk test, and accordingly the Mann-Whitney test was used to test the significance of the association. Correlation analysis was done to quantify the power of association and a further multi-nominal logistic regression analysis was done to identify the demographic predictors for each of the outcomes during the period of the study.

A *p*-value of 0.05 was used as the cutoff point of significance. All analysis was done using IBM SPSS version 24.

## RESULTS

### General demographic characters, sleep, and COVID-related characteristics of the study sample

A total number of 2,474 students filled the questionnaire by the end of the data collection period, constituting the current study’s total sample. The mean age of the study sample was 20.4 years (SD=1.5), with 31.8% being males and 56.8% being urban citizens. About 67% of the study sample were students in non-scientific colleges. Full general demographic characteristics can be found in Table 1. About 7.5% of the study sample had a chronic disease, and about 56% of them had a family member with a chronic disease. About 11.5% of the sample had three or more older people in their household. 45% of the study sample reported already diagnosed sleep problems before COVID-19 and about 63% of them subjectively reported a change in the sleep pattern or qualities after the pandemic. Regarding the direct relation with the pandemic, more than half of the study sample reported no direct relationship with COVID-19 while about 5% reported having the infection themselves or having it in a household member. All details can be found in Table 1.

**Table 1:** Comparisons of the levels of perceived stress, PSQI, and insomnia level among the students. According to the general demographics coffee and smoking consumption (chi-square analysis).

	All respondents (N=2,474)	Low/moderate level of perceived stress (N=1,867)	High level of perceived stress (N=607)	p	Good sleepers (N=511)	Poor sleepers (N=1,963)	p	Sub-clinical insomnia (N=1,699)	Clinical insomnia (N=775)	p
**Gender**										
Male	787 (31.8%)	657 (83.5%)	130 (16.5%)	.000*	200 (25.4%)	587 (74.6%)	.000*	571 (72.6%)	216 (27.4%)	.004*
Female	1,687 (68.2%)	1,210 (71.7%)	477 (28.3%)		311 (18.4%)	1,376 (81.6%)		1,128 (66.9%)	559 (33.1%)	
**Residence**										
Rural	1,068 (43.2%)	826 (77.3%)	242 (22.7%)	.06	215 (20.1%)	853 (79.9%)	.6	748 (70%)	320 (30%)	.2
Urban	1,406 (56.8%)	1,867 (74%)	365 (26%)		296 (21.1%)	1,110 (78.9%)		951 (67.6%)	455 (32.4%)	
**Type of college**										
Medical	578 (23.4%)	419 (72.5%)	159 (27.5%)	.06	130 (22.5%)	448 (77.5%)	.2	388 (67.1%)	190 (32.9%)	.4
Scientific non-medical	235 (9.5%)	178 (75.7%)	57 (24.3%)		49 (20.9%)	186 (79.1%)		164 (69.8%)	71 (30.2%)	
Non-scientific	1,661 (67.1%)	1,270 (76.5%)	391 (23.5%)		332 (20%)	1,329 (80%)		1,147 (69.1%)	514 (30.9%)	
**Academic Year**										
First 2 years	1,191 (48.1%)	942 (79.1%)	249 (20.9%)	.000*	292 (24.5%)	899 (75.5%)	.000*	904 (75.9%)	287 (24.1%)	.000*
3rd year or above	1,283 (51.9%)	925 (72.1%)	358 (27.9%)		219 (17.1%)	1,064 (82.9%)		795 (62%)	488 (38%)	
**Smoking**										
Non-smoker	1,409(56.9%)	1,071 (76%)	338 (24%)	.50	321 (22.8%)	1,088 (77.2%)	.003*	991 (70.3%)	418 (29.7%)	.04*
Smoker	1,065(43.1%)	796 (74.7%)	269 (25.3%)	.002*	190 (17.8%)	875 (82.2%)		708 (66.5%)	357 (33.5%)	
**Caffeine consumption**										
>3 cups	1,983 (80.2%)	1523 (76.8%)	460 (23.2%)		426 (21.5%)	1,557 (78.5%)	.04*	1,384 (69.8%)	599 (30.2%)	.02*
3 or more cups	491 (19.8%)	344 (70.1%)	147 (29.9%)		85 (17.3%)	406 (82.7%)		315 (64.2%)	176 (35.8%)	

Notes: Data presented as frequency and percentage, the

* indicates significance.

### Levels of perceived stress, sleep quality, and insomnia in the study participants

Regarding the level of perceived stress, most of the study sample showed a moderate level of perceived stress (n=1,707, 69%). A quarter of the sample showed a high level of perceived stress (n=607, 24.5%), and the rest showed a low level of stress (n=160, 6.5%).

Regarding sleep quality, only 20.7% of the sample (n=511) were recognized as good sleepers while the rest of the participants (n=1,963, 79.3%) were recognized as bad sleepers or participants who have a bad sleep quality.

Regarding insomnia severity index, most of the sample (n=1,699, 68.7%) scored in the range of sub-clinical insomnia. 43.4% of these were recognized as having no clinically significant insomnia, and 56.6% were recognized as having sub-threshold insomnia. 31.3% of the whole sample had clinical insomnia (n=775), of whom 80.25% had moderate clinical insomnia and 19.75% had severe insomnia.

### Comparisons among the levels of perceived stress, sleep quality, and insomnia and the general demographic characteristics, health, sleep, and COVID-related characteristics

Females had significantly higher frequencies of “high” perceived stress, “poor sleep quality”, and “clinical insomnia” when compared to males. Also, students in the first two years had significantly lower frequencies of “high” level of perceived stress, “poor sleep quality”, and “clinical insomnia” compared to students in their third year or above. Neither type of residence and type of college did not significantly affect any of the three variables of interest. History of smoking (current or ex-smoker) was seen more frequently among poor sleepers and students with clinical insomnia, and having 3 or more cups of caffeine-containing drinks per day was associated with higher frequencies of high level of stress, poor sleep, and clinical insomnia (Table 1). Students who had a chronic disease or a family member with a chronic disease showed significantly higher frequencies of “high” level of stress, “poor sleep quality”, and clinical insomnia compared to those who did not. Also, having 3 or more older household members (more than 50 years) was associated with a higher frequency of “poor sleep quality” and clinical insomnia (Table 2).

**Table 2:** Comparisons of the levels of perceived stress, PSQI, and insomnia level among the students. According to health, sleep quality, and COVID-19 related demographics (chi-square analysis).

	All respondents (N=2,474)	Low or moderate level of perceived stress (N=1,867)	High level of perceived stress (N=607)	p	Good sleepers (N= 511)	Poor sleepers (N=1,963)	p	Sub-clinical Insomnia (N=1,699)	Clinical Insomnia (N=775)	p
**Having a chronic disease**										
Yes	189 (7.6%)	119 (63%)	70 (37%)	.000*	25 (13.2%)	164 (86.8%)	.009*	88 (46.6%)	101 (53.4%)	.000*
No	2,285 (92.4%)	1,748 (76.5%)	537 (23.5%)		486 (21.3%)	1,799 (78.7%)		1,611 (70.5%)	674 (29.5%)	
**Having at least one family member with a chronic disease**										
Yes	1,381 (55.8%)	982 (71.1%)	399 (28.9%)	.000*	223 (16.1%)	1,156 (83.9%)	.000*	852 (61.7%)	529 (38.3%)	.000*
No	1,093 (44.2%)	885 (81%)	208 (19%)		288 (26.3%)	805 (73.7%)		847 (77.5%)	246 (22.5%)	
**Having sleep related problems before COVID-19:**										
Yes	1,119 (45.2%)	736 (65.8%)	383 (34.2%)	.000*	116 (10.4%)	1,003 (89.6%)	.000*	587 (52.5%)	532 (47.5%)	.000*
No	1,355 (54.8%)	1,131 (83.5%)	224 (16.5%)		395 (29.2%)	960 (70.8%)		1,112 (82.1%)	243 (17.9%)	
**Having a change in sleep habits since COVID-19:**										
Yes	1,569 (63.4%)	1,070 (68.2%)	499 (31.8%)	.000*	195 (12.4%)	1,374 (87.6%)	.000*	911 (58.1%)	658 (41.9%)	.000*
No	905 (36.6%)	797 (88.1%)	108 (11.9%)		316 (34.9%)	589 (65.1%)		788 (87.1%)	117 (12.9%)	
**Number of old people in the household:**										
Less than 3	2,191 (88.6%)	1,655 (75.5%)	536 (24.5%)	.8	469 (21.4%)	1,722 (78.6%)	.01*	1,535 (70.1%)	656 (29.9%)	.000*
Three or above	283 (11.4%)	212 (74.9%)	71 (25.1%)		42 (14.8%)	241 (85.2%)		164 (58%)	119 (42%)	

Notes: Data presented as frequency and percentage, the

* indicates significance.

Having a sleep disorder before the COVID-19 pandemic, and reporting a change in the sleep pattern during it, were both associated with significantly higher frequencies of “high” level of stress, poor current sleep quality, and clinical insomnia (Table 2). Regarding the direct relationship to COVID-19, students who had the infection themselves or in a family/household member had significantly higher frequencies of high level of stress, poor sleep quality, and clinical insomnia than those who had no direct relationship to it. Students who knew a relative case that died expressed the highest level of stress while the students, who had the least level of stress, were those who knew a relative case who healed (Table 3).

**Table 3:** Comparisons of the levels of perceived stress, PSQI, and insomnia level among the students. According to COVID-19 related demographics (chi-square analysis).

	All respondents (N=2,474)	Low or moderate level of perceived stress (N=1,867)	High level of perceived stress (N=607)	p	Good sleepers (N=511)	Poor sleepers (N=1,963)	p	Sub-clinical insomnia (N=1,699)	Clinical insomnia (N=775)	p
**Direct relationship to COVID-19 pandemic:**										
No direct relation	1,407 (56.9%)	1,154 (82%)	253 (18%)	.000*	347 (24.7%)	1,060 (75.3%)	.000*	1,068 (75.9%)	339 (24.1%)	.000*
I had/have COVID-19	53 (2.1%)	35 (66%)	18 (34%)		3 (5.7%)	50 (94.3%)		28 (52.8%)	25 (47.2%)	
One member of my family had/have COVID-19	83 (3.4%)	52 (62.7%)	31 (37.3%)		14 (16.9%)	69 (83.1%)		42 (50.6%)	41 (49.4%)	
A friend/relative (non-household) had/have COVID-19	931 (37.6%)	626 (67.2%)	305 (32.8%)		147 (15.8%)	784 (84.2%)		561 (60.3%)	370 (39.7%)	
**Current condition in case of COVID-19 infection:**										
No direct relation	1,324 (53.5%)	1,078 (81.4%)	246 (18.6%)	.000*	325 (24.5%)	999 (75.5%)	.000*	1,017 (76.8%)	307 (23.2%)	.000*
Active (in isolation)	566 (22.9%)	396 (70%)	170 (30%)		92 (16.3%)	474 (83.7%)		331 (58.5%)	235 (41.5%)	
Healed (with or without complications)	283 (11.4%)	218 (77%)	65 (23%)		54 (19.1%)	229 (80.9%)		193 (68.2%)	90 (31.8%)	
Death	301 (12.2%)	175 (58.1%)	126 (41.9%)		40 (13.3%)	261 (86.7%)		158 (52.5%)	143 (47.5%)	

Notes: Data presented as frequency and percentage, the

* indicates significance.

### Relationship between perceived stress, sleep quality, insomnia, and the fear of COVID-19

Table 4 shows that students who had high levels of stress, poor sleep quality, and clinical insomnia had significantly higher scores in the “fear of COVID-19” scale, indicating higher levels of fear than those who had lower levels of stress, good sleep quality, or sub-clinical insomnia, which shows an association between fear of COVID-19 as a separate measure and the stress level or sleep disorders during the period of the study. The overall mean fear of COVID-19 score in the sample was 19.9 (SD=5.8) of a score ranging from 7 to 35 indicating a relatively near high level of fear of COVID-19 in the overall sample. Correlation between the primary outcomes scores and fear of COVID-19 score showed a significant positive fair correlation between fear of COVID-19 and perceived stress score (r=0.4), insomnia score (r=0.4), and PSQI score (r=0.31) (Table 4).

**Table 4:** Comparison between the fear of COVID scores according to the levels of stress, sleep quality and Insomnia severity (Mann-Whitney test).

	Perceived stress		p	Sleep quality		p	Insomnia severity		p
	**Low and moderate**	**high**		**Good sleepers**	**Poor sleepers**		**Sub-clinical**	**Clinical**	
Fear of COVID-19 score	19.0 (5.6)	22.6 (5.5)	.000*	17.3 (5.00)	20.5 (5.8)	.000*	18.6 (5.2)	22.7 (5.9)	.000*

Notes: Data presented as mean (SD), the

* indicates significance.

### Predictors of high stress level, poor sleep quality, and clinical insomnia during the study period identified by regression

For the high perceived stress levels, significant predictors of less “high perceived stress” were; male gender (odds ratio (CI); .49 (.39-.62)), being in the first two academic years (odds ratio (CI); .76 (.62-.93)), not having a chronic disease (odds ratio (CI); .64 (.46-.89)), not having a family member with a chronic disease (odds ratio (CI); .79 (.64-.97)), not having a sleep problem before the pandemic (odds ratio (CI); .52 (.43-.64)), not reporting a sleep change after the pandemic (odds ratio (CI); .38 (.3-.48)), and having less than three caffeine beverages per day (odds ratio (CI); .67 (.53-.84)).

For the sleep quality levels identified by the PSQI score, significant predictors of less “poor sleep” were; male gender (odds ratio (CI); .69 (.55-.86)), being in the first two academic years in college (odds ratio (CI); .74 (.60-.90)), not having sleep problems before the pandemic (odds ratio (CI); .4 (.31-.50)), not reporting a sleep change after the pandemic (odds ratio (CI); .35 (.28-.44)), and being a non-smoker (odds ratio (CI); .78 (.6-.97)).

For the clinical insomnia levels, significant predictors of less “clinical insomnia” were; male gender (odds ratio (CI); .80 (.64-.98)), being in the first two years (odds ratio (CI); .6 (.49-.72)), not having a chronic disease (odds ratio (CI); .45 (.32-.64)), not having a family member with a chronic disease (odds ratio (CI); .72 (.59-.88)), not having sleep disorders before the pandemic (odds ratio (CI); .34 (.28-.41)), not reporting a sleep change after the pandemic (odds ratio (CI); .28 (.23-.36)), and having less than three old people in the household (odds ratio (CI); .68 (0.5-.09)).

## DISCUSSION

COVID-19 pandemic has become a major health issue in the past few months; in this study, we investigated the impact of fear and/or exposure to COVID-19 on anxiety and sleep disturbances among 2,474 students at South Valley University in Egypt. Regarding sleep quality, we found high levels of poor sleep quality and clinical insomnia. Our findings are supported by previous literature in other countries such in Saudi Arabia, where the prevalence of insomnia among the university students and employees in King Saud university during the COVID-19 pandemic was up to 32.2%^4^. In Italy^27^, the Italian lockdown had a significant impact on psychoemotional well-being and sleep where the prevalence of insomnia among university students and administrative staff workers has raised from 15% before COVID-19 to 42% after the lockdown, both in sleep initiation and sleep maintenance insomnia. The level of insomnia among university students, before the COVID-19 era ranged from 9.4% to 38.2% in a systematic review (Jiang et al., 2015) which is higher than the worldwide insomnia prevalence assessed before the pandemic between 3.9% and 22%^24^. Insomnia and poor sleep quality are associated with poorer academic performance (Okano, Kaczmarzyk, Dave, Gabrieli, & Grossman, 2019). In view of all the above, it is expected that students’ productivity would be negatively affected during pandemics and quarantine periods as a result of the associated sleep disturbances.

The second aspect we explored was the perceived stress prevalence amongst students. Our study showed higher levels of moderate and high perceived stress among students compared to the pre-COVID-19 results (93.5% in our study vs. 86.3% in a pre-COVID-19 study)^34^. Likewise results form a Chinese study indicated that higher levels of anxiety were associated with factors related to COVID-19^13^. Also, our results are in line with a recently published study in Saudi Arabia^3^ that evaluated the levels of perceived stress among students in KSA during COVID-19 showing that 85% of the students had moderate or high levels of perceived stress. Wathelet et al. (2020)^38^ found that 24.7% of the university students in France had high levels of perceived stress during the COVID-19 pandemic period, which was comparable to our results. Other studies showed even higher levels of stress compared to our findings, in two studies conducted among Turkish and Polish students, the levels of high perceived stress during the COVID-19 lockdown among students were 71.2% and 56%, respectively^7,33^. These results highlight an important issue in university students during periods of quarantine and pandemics that needs to be addressed. In contrast to these findings, Odriozola-González et al. (2020)^28^ found that the prevalence of moderate to extremely severe symptoms of stress among students and workers of Spanish university in the lockdown period was only 28.14% but this was based on scores from a different scale “depression anxiety stress scale (DASS-21)”, while another study^36^ conducted on B.Sc. nursing students found that only 13.35% of the study participants had high perceived stress levels, which is relatively lower than our findings.

We also found that females had significantly higher levels of perceived stress, poor sleep quality, and clinical insomnia than males. Similar findings were reported in many previous studies^2,3,18,19^.

Having chronic diseases, previous sleep disorder (before COVID-19), change of sleep pattern (after COVID-19), or direct relationship with COVID-19 were significantly associated with higher levels of stress and impaired sleep patterns in this study. A previous study in Morocco reported similar findings on the general population^22^. Also, Yu et al. (2020)^43^ found that worrying about the exposure of a family member to COVID-19 infection was related to sleep disturbances and high stress levels in 30 to 40% of the participants, these results are in line with our findings that students who had more elderly household members during the pandemic, had poorer sleep quality and higher insomnia levels.

To the best of our knowledge, this is the first study to examine sleep quality and insomnia in Upper Egypt during the COVID-19 pandemic period. We included a large sample size of about 2,474 students who filled an online questionnaire voluntarily. However, one important limitation to the findings of this study is the use of convenience sampling through a web survey. This limitation can affect the generalizability of the results; however, the relatively large sample size and the balance in representation among different demographic groups can help make the results more generalizable.

## CONCLUSION

There is an increased insomnia prevalence, poor sleep quality, and high stress level among college students in South Valley University from all majors (medical, scientific non-medical, or non-scientific) during the COVID-19 lockdown. The prevalence is higher in females, older students, and those with chronic diseases, existing sleep disorders or direct relationship with COVID-19 (either themselves or their family members). Fear of COVID-19 was associated with higher levels of stress, clinical insomnia, and poor sleep quality.

### Points of further research

At this point, much further research is warranted such as studies targeting the general population, studies assessing PTSD in the university students, and experimental studies on different interventional therapeutic measures to help students cope effectively with the current situation, especially with the beginning of the new academic year.

## References

[1] Ahorsu DK, Lin CY, Imani V, Saffari M, Griffiths MD, Pakpour AH (2020). The fear of COVID-19 scale: development and initial validation. Int J Ment Health Addict.

[2] Al-Sowygh ZH (2014). Academic distress, perceived stress and coping strategies among dental students in Saudi Arabia. Saudi Dent J.

[3] AlAteeq DA, Aljhani S, AlEesa D (2020). Perceived stress among students in virtual classrooms during the COVID-19 outbreak in KSA. J Taibah Univ Med Sci.

[4] Alfawaz HA, Wani K, Aljumah AA, Aldisi D, Ansari MGA, Yakout SM (2021). Psychological well-being during COVID-19 lockdown: insights from a Saudi State University’s Academic Community. J King Saud Univ Sci.

[5] Altena E, Baglioni C, Espie CA, Ellis J, Gavriloff D, Holzinger B (2020). Dealing with sleep problems during home confinement due to the COVID‐19 outbreak: practical recommendations from a task force of the European CBT‐I Academy. J Sleep Res.

[6] Alyami M, Henning M, Krägeloh CU, Alyami H (2020). Psychometric evaluation of the arabic version of the fear of COVID-19 scale. Int J Ment Health Addict.

[7] Aslan I, Ochnik D, Çınar O (2020). Exploring perceived stress among students in Turkey during the COVID-19 pandemic. Int J Environ Res Public Health.

[8] Auerbach RP, Alonso J, Axinn WG, Cuijpers P, Ebert DD, Green JG (2016). Mental disorders among college students in the World Health Organization world mental health surveys. Psychol Med.

[9] Bastien CH, Vallières A, Morin CM (2001). Validation of the insomnia severity index as an outcome measure for insomnia research. Sleep Med.

[10] Bjorvatn B, Meland E, Flo E, Mildestvedt T (2016). High prevalence of insomnia and hypnotic use in patients visiting their general practitioner. Fam Pract.

[11] Buysse DJ, Reynolds CF, Monk TH, Berman SR, Kupfer DJ (1989). The Pittsburgh sleep quality index: a new instrument for psychiatric practice and research. Psychiatry Res.

[12] Campos-Morales RM, Valencia-Flores M, Castaño-Meneses A, Castañeda-Figueiras S, Martínez-Guerrero J (2005). Sleepiness, performance and mood state in a group of Mexican undergraduate students. Biol Rhythm Res.

[13] Cao W, Fang Z, Hou G, Han M, Xu X, Dong J (2020). The psychological impact of the COVID-19 epidemic on college students in China. Psychiatry Res.

[14] Cohen S, Kamarck T, Mermelstein R (1983). A global measure of perceived stress. J Health Soc Behav.

[15] Cornine A (2020). Reducing nursing student anxiety in the clinical setting: an integrative review. Nurs Educ Perspect.

[16] Araújo FJO, Lima LSA, Cidade PIM, Nobre CB, Rolim Neto ML (2020). Impact of Sars-CoV-2 and its reverberation in global higher education and mental health. Psychiatry Res.

[17] Gaultney JF (2010). The prevalence of sleep disorders in college students: impact on academic performance. J Am Coll Health.

[18] Shiny G, Biju BS (2018). Level of stress and its causes among 1st-year dental students - a cross-sectional study. Natl J Physiol Pharm Pharmacol.

[19] Gualano MR, Lo Moro G, Voglino G, Bert F, Siliquini R (2020). Effects of Covid-19 lockdown on mental health and sleep disturbances in Italy. Int J Environ Res Public Health.

[20] Hawryluck L, Gold WL, Robinson S, Pogorski S, Galea S, Styra R (2004). SARS control and psychological effects of quarantine, Toronto, Canada. Emerg Infect Dis.

[21] Huang Y, Zhao N (2020). Generalized anxiety disorder, depressive symptoms and sleep quality during COVID-19 outbreak in China: a web-based cross-sectional survey. Psychiatry Res.

[22] Idrissi AJ, Lamkaddem A, Benouajjit A, El Bouaazzaoui MB, El Houari F, Alami M (2020). Sleep quality and mental health in the context of COVID-19 pandemic and lockdown in Morocco. Sleep Med.

[23] Kaplan HI, Sadock BJ (1988). Synopsis of psychiatry: behavioral sciences clinical psychiatry.

[24] Kay-Stacey M, Attarian H (2016). Advances in the management of chronic insomnia. BMJ.

[25] Ko CH, Yen CF, Yen JY, Yang MJ (2006). Psychosocial impact among the public of the severe acute respiratory syndrome epidemic in Taiwan. Psychiatry Clin Neurosci.

[26] Lazzari C, Shoka A, Nusair A, Rabottini M (2020). Psychiatry in time of COVID-19 pandemic. Psychiatr Danub.

[27] Marelli S, Castelnuovo A, Somma A, Castronovo V, Mombelli S, Bottoni D (2020). Impact of COVID-19 lockdown on sleep quality in university students and administration staff. J Neurol.

[28] Odriozola-González P, Planchuelo-Gómez Á, Irurtia MJ, Luis-García R (2020). Psychological effects of the COVID-19 outbreak and lockdown among students and workers of a Spanish university. Psychiatry Res.

[29] Onyema EM, Eucheria NC, Obafemi FA, Sen S, Atonye FG, Sharma A (2020). Impact of coronavirus pandemic on education. J Educ Pract.

[30] Pagel JF, Kwiatkowski CF (2010). Sleep complaints affecting school performance at different educational levels. Front Neurol.

[31] Pourhoseingholi MA, Vahedi M, Rahimzadeh M (2013). Sample size calculation in medical studies. Gastroenterol Hepatol Bed Bench.

[32] Rodrigues RND, Viegas CAA, Abreu e Silva AAA, Tavares P (2002). Daytime sleepiness and academic performance in medical students. Arq Neuro-Psiquiatr.

[33] Rogowska AM, Kuśnierz C, Bokszczanin A (2020). Examining anxiety, life satisfaction, general health, stress and coping styles during COVID-19 pandemic in Polish sample of university students. Psychol Res Behav Manag.

[34] Sateia MJ (2014). International classification of sleep disorders - third edition highlights and modifications. Chest.

[35] Seedhom AE, Kamel EG, Mohammed ES, Raouf NR (2019). Predictors of perceived stress among medical and nonmedical college students, Minia, Egypt. Int J Prev Med.

[36] Sheroun D, Wankhar DD, Devrani A, Pv L, Gita S, Chatterjee K (2020). A study to assess the perceived stress and coping strategies among B.Sc. nursing students of selected colleges in Pune during COVID-19 pandemic lockdown. Int J Sci Healthcare Res.

[37] Taylor DJ, Bramoweth AD, Grieser EA, Tatum JI, Roane BM (2013). Epidemiology of insomnia in college students: relationship with mental health, quality of life, and substance use difficulties. Behav Ther.

[38] Wathelet M, Duhem S, Vaiva G, Baubet T, Habran E, Veerapa E (2020). Factors associated with mental health disorders among university students in France confined during the COVID-19 pandemic. JAMA Netw Open.

[39] Wejnert C, Heckathorn DD (2008). Web-based network sampling: efficiency and efficacy of respondent-driven sampling for online research. Sociol Methods Res.

[40] Wejnert C, Pham H, Krishna N, Le B, DiNenno E (2012). Estimating design effect and calculating sample size for respondent-driven sampling studies of injection drug users in the United States. AIDS Behav.

[41] World Health Organization (WHO) (2021). Weekly epidemiological update - 19 January 2021 [Internet].

[42] Xiao H, Zhang Y, Kong D, Li S, Yang N (2020). Social capital and sleep quality in individuals who self-isolated for 14 days during the coronavirus disease 2019 (COVID-19) outbreak in January 2020 in China. Med Sci Monit.

[43] Yu BYM, Yeung WF, Lam JCS, Yuen SCS, Lam SC, Chung VCH (2020). Prevalence of sleep disturbances during COVID-19 outbreak in an urban Chinese population: a cross-sectional study. Sleep Med.

[44] Zhai Y, Du X (2020). Addressing collegiate mental health amid COVID-19 pandemic. Psychiatry Res.

